# Yellow Fever

**Published:** 1878-08

**Authors:** 


### Editorial.

### YELLOW FEVER.

We have before us a reprint from the Transactions of the Medical Association of Georgia, on yellow fever, its cause and treatment, by Dr. J. C. LeHardy, of Savannah, Georgia.

We are pleased to see such an able production from one present during the violent epidemic of the disease in 1876. Ingenious theories, however false they may be, are likely to impress the reader, and practically work serious injury to the public. This, we firmly believe, has been the case for many years in regard to the origin and cause of yellow fever. The idea of importation seems to have possessed the people generally since its first appearance in this country. The efforts, therefore, of municipal authorities, health officers, etc., have been expended in the wrong direction and uselessly.

Dr. LeHardy's patient, close observation of its ravages in Savannah, enables him to give something more than vague, unsustained theory. His narration of facts bearing on the subject of origin will, at any rate, excite doubts as to the correctness of the importation theory, and tend to the abatement of the local cause. This will doubtless lead to more beneficial results than the most vigorous system of quarantine that can be adopted. The doctor strikes upon the true cause of the disease in the marshes and pools on the outskirts of the city, and in calling attention to them as giving origin to the fever, will do more to protect the inhabitants against its ravages than the health officer and the whole United States navy can by quarantining. Dr. LeHardy's labor is not directed to the maintenance of a wild theory, void of practical and useful results, but to the adoption of means which will probably prevent the most deadly scourge to which Savannah is subject.

We are gratified to see that in New Orleans, where the fever is now raging, attention is being directed to the true origin of the malady. It is said: "the filthy condition of the city is owing partly to heavy rains forming stagnant pools on the low grounds," etc. "The board of health is censured for allowing the fever to spread."

If all boards of health would depend more on preventing stagnant pools, than frustrating commerce and travel by nonsensical quarantine, the people would be much better protected against yellow fever.

